# Scalding by Steam

**Published:** 1836

**Authors:** 


### IV.—Scalding by Steam.

Hot water scalds through our apparel, even worse than when it is thrown on the naked surface, for it is kept longer in contact with the part, and evaporation being prevented, the process of cooling cannot commence so soon. But does hot steam injure us through our clothing? This question was lately answered in a very conclusive, but melancholy manner, on board one of our steam-boats. The Flora, on an upward voyage, 40 or 50 miles below this city, had the equalization pipe of her boilers fractured, and a copious current of steam made its escape. The accident happened before day, when most of the passengers were asleep in their berths. The cabin door being open at the time, the steam rushed in, and the inmates, on awakening, found themselves enveloped in this new atmosphere. Many of them had the presence of mind to bury up their heads and lie still; but a greater number, in alarm and confusion of mind, sprang out of bed; and in attempting to make their way to the cabin door, encountered the entering columns. Twelve or fourteen were thus scalded in various degrees—one of whom died almost immediately, and three or four others at different periods afterwards. We had an opportunity of seeing the whole of these patients, and a majority of them were brought to the Cincinnati Hospital. In every case, those parts of the body only were scalded, that were uncovered—such as the face, neck, shoulders, hands, feet and legs below the knees. One of the patients was scalded to his hips. He had left his berth without pantaloons or drawers. We had not an opportunity of ascertaining, decisively, that linen constituted a sufficient protection, but of the perfect efficacy of flannel there was no doubt. We would suggest then to steam-boat voyagers, the safety, (as well as decency) of sleeping in flannel; and of continuing in their berths with their heads covered up, under accidents that may fill the cabin with steam. In a brief period indeed, its temperature will be so reduced, as to render it harmless. We thought it an object of interest, to inquire whether any of these unfortunate persons were scalded in the mouth, throat, nostrils or lungs; but nothing indicated any injury of the mucous surfaces; and we have the impression, that they are protected from the action of hot steam by their secretion. The lips and noses of several were scalded, up to the edges of the mucous membranes but no further.

